# Degradable Water-Swellable Elastomers from Biobased Deep Eutectic Monomers

**DOI:** 10.1021/acssuschemeng.5c13563

**Published:** 2026-03-31

**Authors:** Lucila Navarro, Matías D. Hartman, Sebastian Locatelli, Santiago E. Vaillard, Matías L. Picchio, Roque J. Minari, Haritz Sardon, David Mecerreyes

**Affiliations:** † Group of Applied Organic Chemistry, Instituto de Desarrollo Tecnológico para la Industria Química (INTEC), CCT-Santa Fe, CONICET-UNL, Colectora Ruta Nacional. 168, Km 1, Paraje EL Pozo, Santa Fe 3000, Argentina; ‡ Facultad de Bioquímica y Ciencias Biológicas, Universidad Nacional del Litoral (UNL), Edificio FBCB, Ciudad Universitaria UNL, C.C. 24, Santa Fe S3000ZAA, Argentina; § Group of Polymers and Polymerization Reactors, Instituto de Desarrollo Tecnológico para la Industria Química (INTEC), CCT-Santa Fe, CONICET-UNL, Colectora Ruta Nacional. 168, Km 1, Paraje El Pozo, Santa Fe 3000, Argentina; ∥ POLYMAT, Department of Mining-Metallurgy Engineering and Materials Science, School of Engineering, University of the Basque Country (UPV/EHU), Plaza Torres Quevedo 1, Bilbao 48013, Spain; ⊥ IKERBASQUE, Basque Foundation for Science, Plaza Euskadi 5, Bilbao 4009, Spain; # POLYMAT, Department of Polymers and Advanced Materials: Physics, Chemistry and Technology, Faculty of Chemistry, University of the Basque Country UPV/EHU, Paseo Manuel de Lardizábal, 3, Donostia-San Sebastián 20018, Spain; ∇ POLYMAT, University of the Basque Country UPV/EHU, Avenida Tolosa 72, Donostia-San Sebastián, Gipuzkoa 20018, Spain

**Keywords:** polymerizable eutectic solvents (PDES), itaconic acid, succinic acid, ionic diols, polyDES elastomers, antimicrobial activity

## Abstract

Deep eutectic monomers (DEMs) are emerging as a versatile platform for designing functional polymeric materials directly from eutectic mixtures that can act as polymerizable building blocks. Here, we report a new family of DEMs derived from choline chloride (ChCl)–based ionic diols and biosourced diacids, including itaconic and succinic acids, enabling the formation of degradable cationic polyester elastomers. The introduction of an additional hydroxyl group into the ionic monomer promotes efficient polycondensation after eutectic formation, allowing solvent-free synthesis under mild conditions. By systematically varying the alkyl chain length of the ionic diol and the ratio of unsaturated to saturated diacids, we establish how subtle changes in monomer architecture dictate network topology and, consequently, macroscopic properties. The resulting water-swellable elastomers exhibit tunable cross-linking densities, chain mobility, and swelling behavior, along with marked differences in ionic conductivity. Moreover, the ability to integrate biodegradability into cationic architectures marks a significant step toward transient, resorbable, and biologically compatible materials, opening opportunities in tissue scaffolding, wound care, and therapeutic delivery. In addition, the networks exhibit intrinsic antimicrobial activity, which can be maximized through molecular design, particularly in formulations with a higher unsaturated acid content that promotes chain mobility and cationic site exposure. Overall, this work establishes eutectic monomer engineering as a powerful and sustainable route to biodegradable, antimicrobial, and ionically conductive elastomers, expanding the chemical space of functional materials accessible from deep eutectic solvents (DES)-derived monomers.

## Graphical abstract

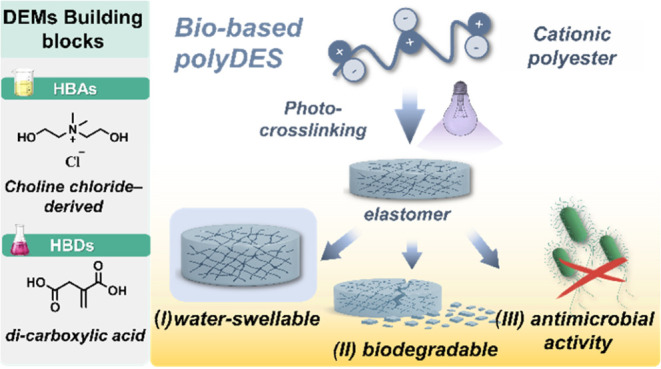

## Introduction

Deep eutectic solvents (DESs) have emerged as a new generation of sustainable solvents and functional additives that align with the principles of green chemistry. DES are typically formed by combining a quaternary ammonium or phosphonium salt with a hydrogen bond donor (HBD), usually selected from inexpensive and safe compounds. In these systems, the salt acts as the hydrogen bond acceptor (HBA), while the donor molecule interacts through strong hydrogen bonding with the halide anion. This interaction leads to charge delocalization and an abnormal depression of the freezing point of the mixture compared to that of the ideal solution. DESs share many physicochemical features with conventional ionic liquids (low volatility and nonflammability); however, they offer additional advantages such as low cost and straightforward preparation without the need for purification.^1^

Beyond their traditional role as solvents,^2–4^ DESs can also act as reactive precursors, giving rise to deep eutectic monomers (DEMs) when at least one eutectic component bears polymerizable groups.^5^ As an advantage, the formation of liquid monomers enabled the use of mild polymerization techniques, such as photopolymerization and polycondensation, for the synthesis of new polymeric materials. This feature is particularly relevant because the liquid nature of the monomers eliminates the need for additional solvents, ensuring better miscibility between components and facilitating homogeneous reaction media. The mild conditions reduce the risk of thermal degradation or unwanted side reactions, which is especially advantageous when working with sensitive functional groups or biobased precursors. As an example of photopolymerization, DESs have been prepared from choline chloride (ChCl) combined with acrylic or methacrylic acid monomers, followed by frontal polymerization of the monomer, with the added advantage that ChCl can be recovered and recycled after the reaction.^6,7^ Another reported approach involves the use of quaternary amines functionalized with acrylate groups as HBAs together with polyphenols as HBDs, broadening the range of DEM formulations available for polymer synthesis.^8^ In the case of polycondensation, only a limited number of examples have been reported. These typically rely on the use of tri- or dicarboxylic acids (e.g., citric acid) in combination with quaternary amine derivatives acting as polyols.^5^

Among the HBAs, ChCl is one of the most widely used for the preparation of DESs due to its low cost and biocompatibility.^9–11^ As a quaternary ammonium salt, it can readily establish strong hydrogen-bonding interactions with a variety of HBDs, such as carboxylic acids,^12,13^ polyols,^14^ or amides,^15^ leading to the formation of stable eutectic mixtures. A key advantage of ChCl lies in its origin, as it is a naturally occurring compound and an essential nutrient, which makes DESs based on this salt environmentally friendly and suitable for applications where safety is a concern. Furthermore, ChCl-based DESs exhibit low volatility, nonflammability, and high solvation ability properties that have promoted their use in diverse fields, including polymer synthesis,^16^ electrochemistry,^17^ and biomass processing.^18^ These characteristics make ChCl an ideal candidate for the design of functional eutectic monomer systems for the development of advanced polymeric materials.

Among the different organic acids available as HBDs for DES design, itaconic, maleic, and fumaric acids stand out as biobased precursors.^3,19^ Their structures offer both carboxylic and vinyl functionalities, enabling polycondensation reactions while retaining reactive vinyl groups for further radical cross-linking.^20–22^ In combination with succinic acid and another bioderived diacid, the network architecture and cross-linking density can be systematically tuned.^22^ The use of hydroxyl-functionalized ChCl-derived ionic monomers with dicarboxylic acids through DES formation and subsequent polycondensation could enable the preparation of a new class of bioabsorbable cationic polyesters. These polymers combine the advantages of DES chemistry-simplicity, sustainability, and versatility with the functionality of cross-linkable polyesters. When further cross-linked by UV irradiation, these polyDES could provide networks with tunable mechanical, thermal, and swelling properties, as well as adjustable biodegradability and ionic conductivity.

Recent years have witnessed significant progress in the development of biobased polyester elastomers with controlled and tunable degradation behavior.^23^ Elastomers derived from renewable diacids and diols have attracted increasing attention due to their inherent hydrolytic degradability, biocompatibility, and structural versatility, which allow degradation rates to be tailored through molecular design, cross-link density, and network architecture.^24^ Strategies, such as the incorporation of multifunctional cross-linkers, dynamic covalent bonds, or heterogeneous network structures, have enabled the combination of mechanical robustness with controlled degradation under physiological or environmental conditions. Comprehensive reviews have highlighted that, beyond traditional natural rubber systems, synthetic biobased elastomers, including polyesters, polyurethanes, and ionically modified networks, represent a promising platform for sustainable and degradable elastomeric materials, although the integration of degradability into cationic elastomer architectures remains comparatively underexplored.^25,26^

In this work, we introduce a new family of deep eutectic monomers (DEMs) derived from biobased diacids, namely, itaconic and succinic acids, and tailored ChCl derivatives. To impart reactivity toward polyester formation, the ChCl-based ionic monomers were strategically engineered to incorporate an additional hydroxyl group, enabling them to function as diols and undergo controlled polycondensation with dicarboxylic acids once the eutectic mixture is formed. By systematically varying both the alkyl chain length of the ionic monomer and the ratio of unsaturated to saturated acids, we establish how subtle changes in the eutectic architecture translate into precise control over the molecular organization and bulk properties of the resulting elastomeric networks. In addition, the quaternary ammonium groups embedded within the polymer backbone impart a strong affinity for water, enabling the elastomers to absorb large amounts of water and undergo substantial swelling. Moreover, the incorporation of hydrolytically labile polyester segments into a cationic network, an uncommon combination in synthetic polymer design, introduces a well-defined and tunable degradation pathway, a key requirement for transient, resorbable materials intended for biological environments. Coupled with the inherent antimicrobial activity of the cationic backbone, these attributes position the resulting elastomers as promising candidates for next-generation biomedical technologies.

## Experimental Section

### Materials

All chemicals were used without further purification. Dimethylethanolamine, 2-chloroethanol, 3-chloro-1-propanol, 6-chloro-1-hexanol, and 8-chloro-1-octanol were purchased from TCI Chemicals. Itaconic acid, succinic acid, maleic acid, fumaric acid, hydroquinone (HQ), *p*-toluenesulfonic acid (*p*TSA), darocur 1173 (2-hydroxy-2-methylpropiophenone), and acrylic acid were purchased from Sigma-Aldrich.

### Synthesis of Ionic Monomers

A series of ionic monomers was synthesized via quaternization of dimethylethanolamine with alcohol chlorides of varying alkyl chain lengths, specifically, 2-chloroethanol, 3-chloro-1-propanol, 6-chloro-1-hexanol, and 8-chloro-1-octanol. The reactions were carried out in a round-bottomed flask equipped with a reflux condenser under an argon atmosphere. The mixtures were maintained at 70 °C for 24 h to complete the reaction. Following completion, the products were purified by recrystallization from hot ethanol and subsequently washed with ethyl acetate. All of the resulting compounds were isolated as white crystalline powders.

### Synthesis of PolyDES

The synthesis of polyDES prepolymers was carried out by combining 1 equiv of the ionic monomer with varying ratios of itaconic acid and succinic acid, maintaining a total of 1 equiv of dicarboxylic acid. The reaction mixture was placed in a round-bottom flask previously purged with argon. Hydroquinone (HQ) was introduced as a radical scavenger to preserve the vinyl groups of itaconic acid during the polycondensation, while *p*-toluenesulfonic acid (pTSA, 0.1 mol % relative to itaconic acid) was employed as the catalyst. After DES formation at 90 °C, the temperature was increased to 120 °C for 24 h to accelerate the reaction time. Afterward, the system was subjected to vacuum at the same temperature for an additional 8 h to complete the process.

### Elastomer Preparation and Cross-Linking

About 200 mg of the prepolymer was mixed with 30 wt % of acrylic acid (57.1 μL), 20 wt % of deionized water (40 μL), and 5 wt % of Darocur 1173 (9.2 μL) as the photoinitiator. The mixture was cast onto a silicone plate and subsequently exposed to UV irradiation. Photocuring was performed using a setup equipped with six 9 W UV lamps (0.18 W cm^–2^), positioned 6 cm above the films, for a defined exposure time.

### Characterization Techniques

#### ^1^H NMR

Proton nuclear magnetic resonance (^1^H NMR) spectra (300 MHz) were obtained with a Bruker Avance II spectrometer (Karlsruhe, Germany). Measurements were performed for monomers, prepolymers, and cross-linked films, using deuterium oxide (D_2_O) as solvent. Solvent signal suppression was achieved using the WATERGATE pulse sequence.

#### Infrared Spectroscopy

Fourier transform infrared spectra were obtained with a Shimadzu 8201 PC apparatus (Kyoto, Japan) under attenuated total reflectance (ATR) mode, in the range of 400–4000 cm^–1^.

#### Gel Point Determination Based on Chain Mobility

The gel point was determined by applying the Carl–Purcell–Meiboom–Gill (CPMG) pulse sequence using a Time Domain NMR Analyzer (Spin Track, Resonance Systems, GmbH, Germany). Each experiment was carried out with 150 scans, 1200 echoes, and a relaxation delay of 5 s. The obtained decay curves were fitted to a biexponential function according to eq 1

1 $$I\left(t\right)=a_{01}\;\exp\left(-\frac{t}{T_{21}}\right)+a_{02}\left(\frac{t}{T_{22}}\right)+y_{0}$$

where *T*_2_ corresponds to the proton relaxation times, and *I* corresponds to the signal intensity, associated with the soft and rigid polymer domains, respectively. The evolution of *T*_22_ as a function of cross-linking time was analyzed by bilinear regression, and the intersection point of the two fitted lines was taken as the gel point.

#### Water Vapor Uptake

Prior to the swelling experiments, the film samples were dried at 50 °C for 4 days to ensure a constant weight. Samples were placed in a controlled humidity chamber, ensured with 75% humidity. At predetermined time intervals, the samples were weighed. The moisture content was determined gravimetrically according to eq 2

2 $$water\;vapor\;uptake\;\left[\%\right]=\frac{W_{h}-W_{0}}{W_{0}}\times 100$$

where *W*_h_ is the hydrated mass, and *W*_0_ is the initial dry mass. This assay was performed in triplicate for each sample.

#### Swelling Index

Prior to the swelling experiments, the film samples were dried at 50 °C for 4 days to ensure constant weight. Afterward, the specimens were cut into discs of 10 mm in diameter and 1 mm in thickness and immersed in deionized water. At predetermined time intervals, the samples were withdrawn; the excess surface water was gently removed with absorbent paper, and the specimens were weighed. The swelling index was then calculated according to eq 3

3 $$swelling\;index\;\left[\%\right]=\frac{W_{s}-W_{0}}{W_{0}}\times 100$$

where *W*_s_ is the wet mass, and *W*_0_ is the initial dry mass. This assay was performed in triplicate for each sample.

#### Gel Content

Gel content was measured on films exposed to UV light for 60 min. Samples were cut into disc-shaped films of 0.7 cm in diameter and 1 mm in thickness. The samples were dried at 50 °C for 3 days, weighed, and then placed in water at 50 °C for 2 days to ensure that all unreacted materials were eliminated. Afterward, the samples were left to dry for 2 days at 50 °C. Gel contents were obtained gravimetrically, according to eq 4

4 $$\mathrm{gel}\;\mathrm{content}\;\left[\%\right]=\frac{m_{f}}{m_{i}}\times 100$$

where *m*_i_ and *m*_f_ are the initial and final masses of cross-linked polymer film, respectively. The experiments were conducted 4 times per sample.

#### Low-Field (LF) NMR Relaxation Measurements

Transverse relaxation times (*T*_2_) were determined for both dry and hydrated elastomers by time-domain NMR using a Spin Track analyzer (Resonance Systems GmbH, Germany). Measurements were carried out with the CPMG pulse sequence, employing a 90–180° pulse spacing of 8 μs. Each acquisition consisted of 150 scans and 1200 echoes, with a 5 s relaxation delay between successive scans.

#### Proton Multiple Quantum NMR

^1^H Multiple Quantum (MQ) NMR measurements were performed on a Spin Track Time Domain NMR analyzer. About 90° pulses were set to 5 μs, and 6 scans were recorded for each sample in a dry state. Double quantum (DQ) build-up curves were obtained after proper normalization. A long effective *T*_2_ relaxation time results in a prolonged tail, which can be fitted and subtracted.^27^ Following tail removal, relaxation effects were corrected by applying a point-by-point normalization to the DQ build-up curves using the sum function.

5 $$I_{\mathrm{nDQ}}\left(t_{\mathrm{DQ}}\right)=I_{\mathrm{DQ}}\left(t_{\mathrm{DQ}}\right)/\left(I_{\sum \mathrm{MQ}}\left(t_{\mathrm{DQ}}\right)-\mathrm{tail}\right)$$

where *I*_nDQ_ is the normalized DQ intensity, *I*_DQ_ is the DQ intensity build-up, and the *I*_ΣMQ_ is the MQ sum intensity.

#### Tensile Tests

Tensile tests were conducted at ambient temperature by using an Instron 3344 universal testing machine fitted with a 10 N load cell. Dog-bone-shaped specimens were prepared with a gauge length of 25 mm, a width of 5 mm, and a thickness of 1 mm in the elongation section. For each material, three specimens were tested at a constant crosshead speed of 10 mm·min^–1^ until fracture. The experimental stress–strain data were analyzed by using the Neo–Hookean hyperelastic model for isotropic and incompressible systems, in which the shear modulus (*G*) serves as the sole fitting parameter. According to this model, the relationship between applied stress (σ) and the stretch ratio (λ = *L*(*t*)/*L*_0_) is described by eq 6.

6 $$\sigma =G\left(\lambda^{2}-\frac{1}{\lambda }\right)$$

The strain is defined as the ratio of the sample change in length to the undeformed length, as ϵ = λ – 1.

Cross-linking densities (*n*) were estimated based on the theory of rubber elasticity according to eq 7

7 $$n=\frac{G}{3\mathrm{RT}}$$

where *n* is the number of network chain segments per unit volume [mol L^–1^], *R* is the universal gas constant (8.3144 J mol·K^– 1^), and *T* is the temperature [K].

#### Thermal Properties

Thermal stability was assessed by thermogravimetric analysis (TGA) using TGA Q500 (TA Instruments) equipment. Temperature scans were performed at a rate of 10 °C min^–1^ from room temperature to 600 °C. The glass transition temperature (*T*_g_) of elastomers was measured by differential scanning calorimetry (DSC Q2000, TA Instrument) at a heating rate of 10 °C·min^–1^. The cured films were evaluated in a temperature range from −50 °C up to 100 °C to avoid undesired thermal degradation of the samples. DSC and TGA experiments were performed under an inert N_2_ atmosphere (flow: 40 mL min^–1^).

#### Scanning Electron Microscopy (SEM)

Film samples were examined using a Dual Beam SEM (Zeiss CrossBeam 350). Images were collected from both the surface and the cross-sectional areas of the films. For cross-sectional analysis, the specimens were mechanically cut using a precision cutter to obtain clean fracture surfaces suitable for observation.

#### Conductivity

Electrochemical impedance spectroscopy (EIS) was carried out by using a PalmSense4 potentiostat/galvanostat to evaluate the ionic conductivity of the ionic elastomers. The samples, shaped as circular disks, were placed between two stainless steel electrodes with an active area of 0.503 cm^2^. Measurements were recorded at room temperature across a frequency range of 1 × 10^5^–1 Hz by applying an AC perturbation of 10 mV.

#### Degradation Analysis

*In vitro* degradation studies were performed gravimetrically. Only the gel part of the materials was evaluated, so prior to the degradation experiments, the samples were incubated in warm water and then dried at 50 °C for 3 days. Disk-shaped films from cured polymer samples (5 mm in diameter and 1 mm thick) were immersed in PBS buffer at 37 °C for a period of time, after which the samples were withdrawn from the immersion medium, washed 3 times with deionized water, and left to dry for 48 h at 50 °C for final weighing. Mass losses were calculated according to eq 8

8 $$mass\;loss\;\left[\%\right]=\frac{\left(M_{0}-M_{p}\right)}{M_{0}}\times 100$$

where *M_p_* is the dry mass at a period of time *p*, and *M*_0_ is the initial dry mass.

#### Antimicrobial Study

*Escherichia coli* cells from the DH5α strain were grown overnight in Lysogeny Broth (LB) media at 37 °C and constant shaking of 200 rpm. Afterward, bacteria solution was adjusted to OD_600_ = 1, and 1 mL of the solution was added to 1.5 mL microcentrifuge tubes containing a 3 mm-diameter sample. As a negative control, a tube without film was also included. All tubes were incubated at room temperature with slow shaking (40 rpm), a condition where no significant growth will occur during the experiment. After 4 h, 100 μL of the samples were collected, and serial dilutions were made in fresh LB media before plating on LB agar plates. After overnight incubation at 37 °C, colony-forming units (CFU) were counted. Each test was carried out in three replicates.

## Results and Discussion

In this work, we introduce a straightforward strategy to design functional elastomers, starting from eutectic mixtures. Unlike conventional approaches, the process exploits the ability of simple hydrogen-bonded monomer mixtures of ChCl derivatives and dicarboxylic acids to form liquid monomers upon mild heating to design functional elastomers. This new family of DEMs can be directly polymerized into biodegradable polyDES by a polycondensation reaction. The subsequent photo-cross-linking step, carried out in the presence of acrylic acid, a small amount of water, and a photoinitiator, yields a robust cationic elastomer network with the potential to absorb a great amount of water due to the presence of the quaternary ammonium groups. As summarized in Figure 1a, this stepwise transformation from solid precursors to DES, then to polyDES, and ultimately to a cross-linked network underscores the potential of DEM-based chemistry as a sustainable and versatile route for the preparation of degradable cationic polyester elastomers.

**Figure 1:**
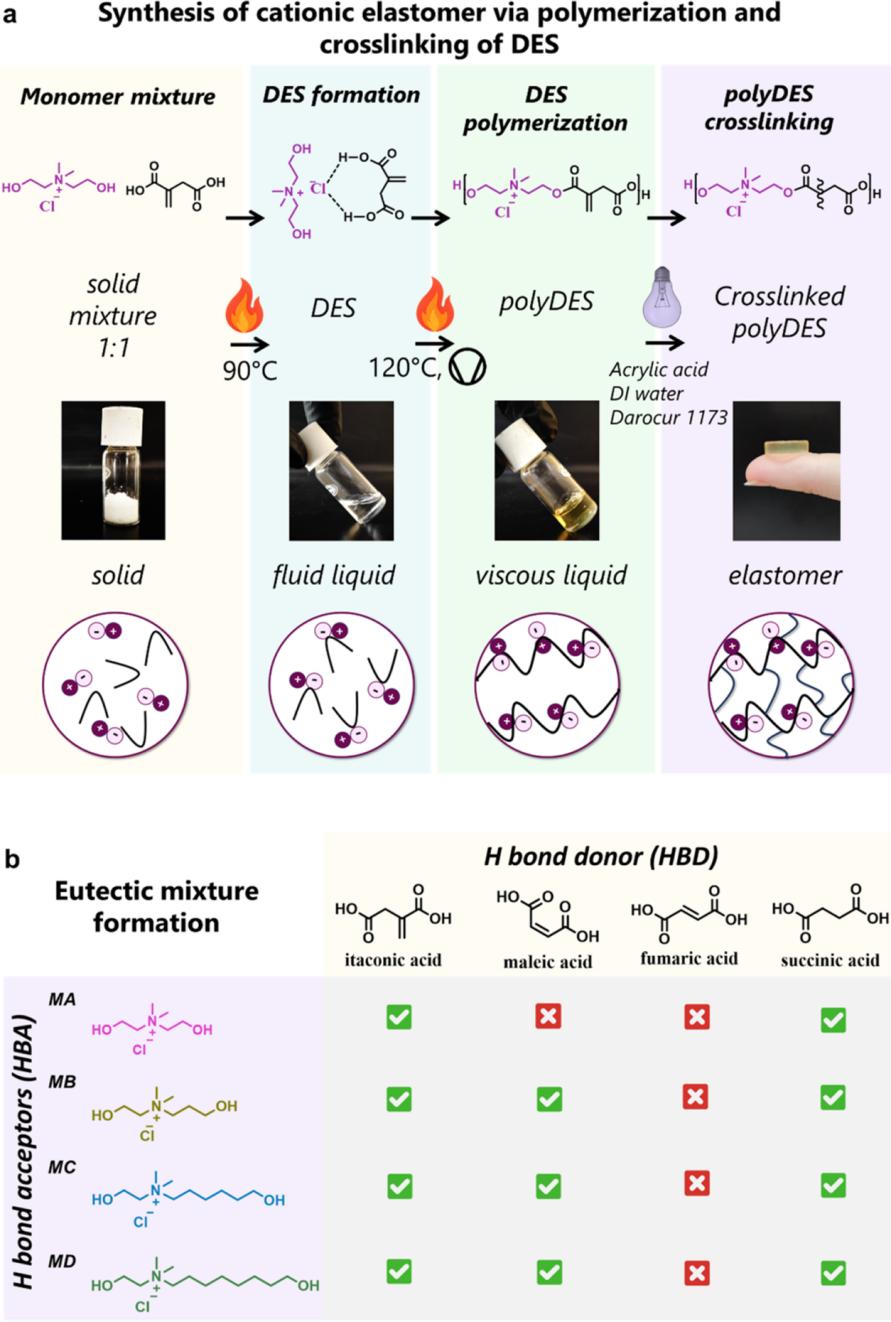
(a) Schematic illustration of the preparation of cross-linked cationic polyesters by polymerizing and cross-linking of DES. (b) Eutectic mixture formation between the obtained ionic monomers as HBAs and different dicarboxylic acids as HBDs, in a 1:1 ratio.

First, a series of ChCl biobased monomers was synthesized through quaternization reactions between dimethylethanolamine and alcohol chlorides bearing alkyl chains of different lengths (Figure S1a, Supporting Information, SI). The objective of this step was to generate bifunctional diol monomers containing two hydroxyl groups, enabling their subsequent step-growth polycondensation with a dicarboxylic acid monomer. The successful formation of these monomers was confirmed by ^1^H NMR spectroscopy, as shown in Figure S1b. To evaluate their ability to form eutectic mixtures, four dicarboxylic acids (itaconic, succinic, maleic, and fumaric acids) were tested in combination with the synthesized ionic monomers in a 1:1 molar ratio, as stoichiometry is needed to form a polymer in a subsequent polycondensation reaction. As summarized in Figure 1b, itaconic and succinic acids successfully formed highly stable liquid eutectic mixtures at room temperature with all monomers, whereas fumaric acid did not yield any eutectic mixtures. Maleic acid, in turn, displayed intermediate behavior, producing eutectic mixtures with all monomers except the one bearing the shortest alkyl chain. This selective behavior may be associated with differences in the steric and geometric constraints of the dicarboxylic acids, particularly the *cis–trans* configuration in maleic and fumaric acids.

Among the four derivatives obtained, the monomers with the shortest (monomer A, MA) and longest alkyl chains (monomer D, MD) were selected for the next polymerization step. This selection aimed to investigate the effect of alkyl chain length on the ionic density per unit mass of the polymer. Then, itaconic and succinic acids were chosen as HBDs to prepare prepolymers, as they were the two carboxylic acids that were able to form eutectic mixtures with the chosen HBAs. By varying the copolymerization ratio (saturated/unsaturated acid), we could control the final cross-linking density.

### PolyDES Synthesis and Characterization

Two polyDES families were synthesized via polycondensation of the bifunctional monomers bearing the shortest alkyl chain (MA; polyDES I) or the longest alkyl chain (MD; polyDES II), as illustrated in Figure 2a. The corresponding monomer feed ratios are summarized in Table S1. The reaction was conducted at 120 °C under vacuum, *p*-toluenesulfonic acid (*p*TSA) was used as a catalyst, and hydroquinone (HQ) was used as a radical scavenger to prevent premature consumption of the vinyl groups.

**Figure 2:**
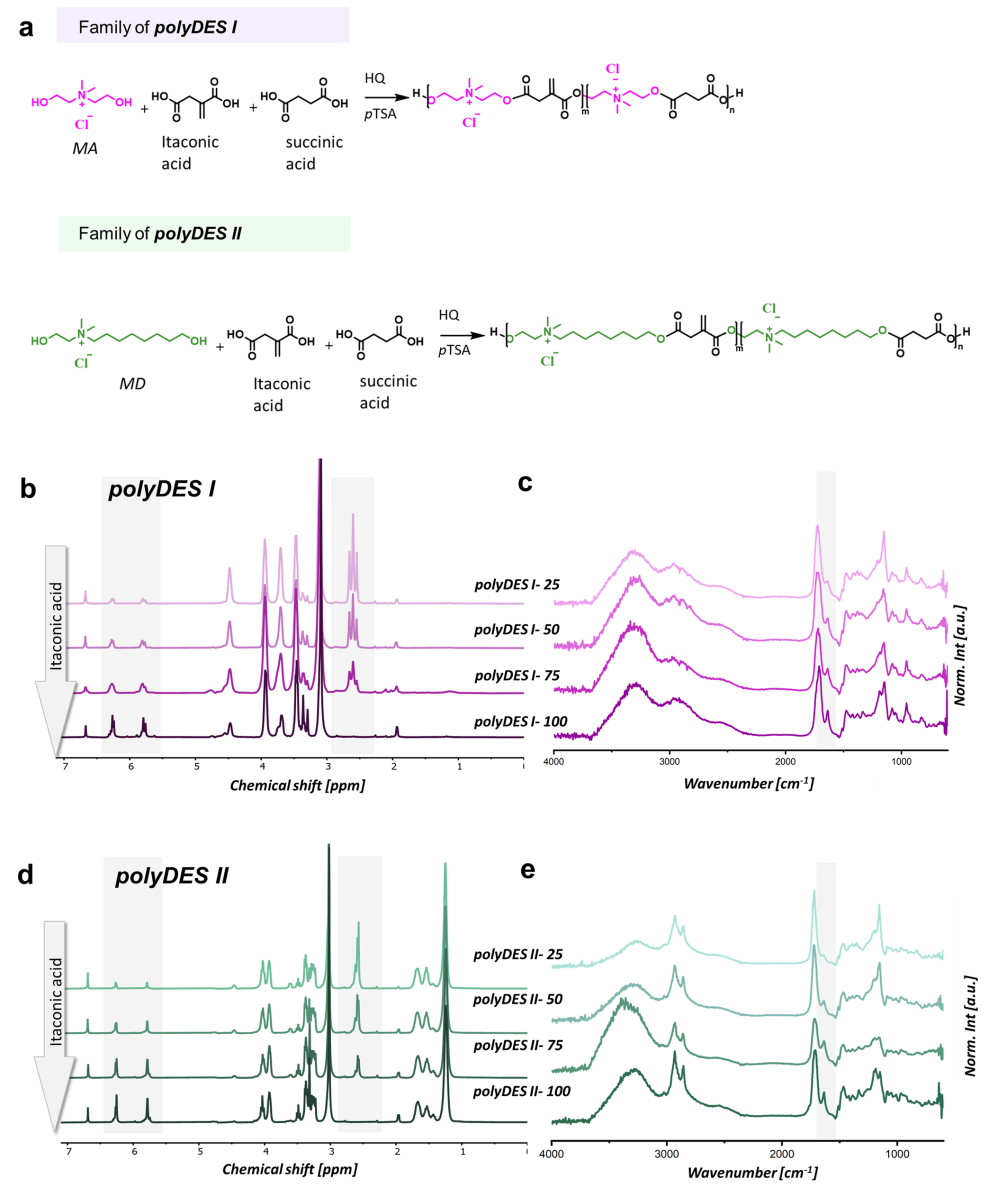
(a) Synthesis of cationic polyester polyDES families. (b) ^1^H NMR of polyDES I. (c) FTIR of polyDES I. (d) ^1^H NMR of polyDES II. (e) FTIR of polyDES II. The gray-highlighted region in NMR spectra, from 5.5 to 6.5 ppm, corresponds to the protons of the double bond (−C=CH_2_) from itaconic acid, and the region from 2.5 to 3.0 ppm corresponds to the protons of the aliphatic segment from succinic acid (−CH_2_CH_2_−). The highlighted region in FTIR spectra, at 1660 cm^–1^, corresponds to the C=C bond stretch.

Structural characterization of the resulting polyDES was performed by ^1^H NMR (left panels) and FTIR (right panels) spectroscopy to confirm the chemical structure of the cationic polyester. Figure 2b,d displays the ^1^H NMR spectra for polyDES I and polyDES II, respectively. The highlighted region between 5.5 and 6.5 ppm corresponds to the vinyl protons of the itaconic acid double bond (−C=CH_2_). As expected, the highest signal intensity was detected in the prepolymers, with the largest proportion of itaconic acid. This trend is consistent with the FTIR spectra as shown in Figure 2c,e, where the highlighted regions correspond to the characteristic C=C stretching vibrations. In addition, the appearance of resonances between 2.5 and 3.0 ppm in the ^1^H NMR spectra indicates the incorporation of succinic acid into the prepolymer structure, assigned to the protons of the aliphatic −CH_2_–CH_2_– segment. Given that neither of the CH_2_ group of succinic acid nor the CH_2_ group of itaconic acid is resolved in the ^1^H NMR spectra, regardless of whether these monomers are part of the polymer chain or are the terminal groups, it was not possible to estimate the molecular weights of the polyesters by end group analysis. However, at least for polyDES I, CH_2_ groups of the succinic acid in the 2.5–3.0 ppm range appear as clearly resolved triplets, indicating that the two methylene groups α to the carboxylic groups are non-equivalents. This observation suggests that the succinic acid monomers are either terminal or located near the end of the polymer chain, implying low molecular weights, which were, in fact, expected. The same lack of resolution in the ^1^H NMR spectra of the CH_2_ groups for both succinic and itaconic acids precludes copolymer/homopolymer composition analysis.

### Evolution of the Cross-Linking Process in PolyDES Systems under UV Irradiation

To enhance the reactivity and tailor the final properties of the networks, polyDES families I and II were copolymerized with 30 wt % acrylic acid, in the presence of 20 wt % of deionized water and with 5 wt % of Darocur 1173, as photoinitiator (all quantities were calculated with respect to the polyDES mass). A schematic representation of the cross-linking process is shown in Figure 3a. The introduction of acrylic acid provides additional vinyl groups with high radical reactivity, thereby accelerating the photocuring process and enabling the formation of more densely cross-linked networks. Moreover, the incorporation of acrylic acid contributes to the modulation of mechanical and swelling properties by increasing the number of carboxylic functionalities, which enhances the hydrophilicity and water uptake. Although the formulations contain 20 wt % of added water during the photocuring step, this small amount of water is only incorporated to reduce viscosity, homogenize the mixture, and enable proper film preparation. Importantly, this water fraction does not confer hydrogel-like behavior. After drying the films and subsequently exposing them to a humid environment (75% relative humidity), the materials did not reabsorb a substantial amount of water beyond their initial value, demonstrating that their equilibrium water content is intrinsically low and dictated by ambient hydration rather than by significant swelling during their preparation (Figure S2).

**Figure 3:**
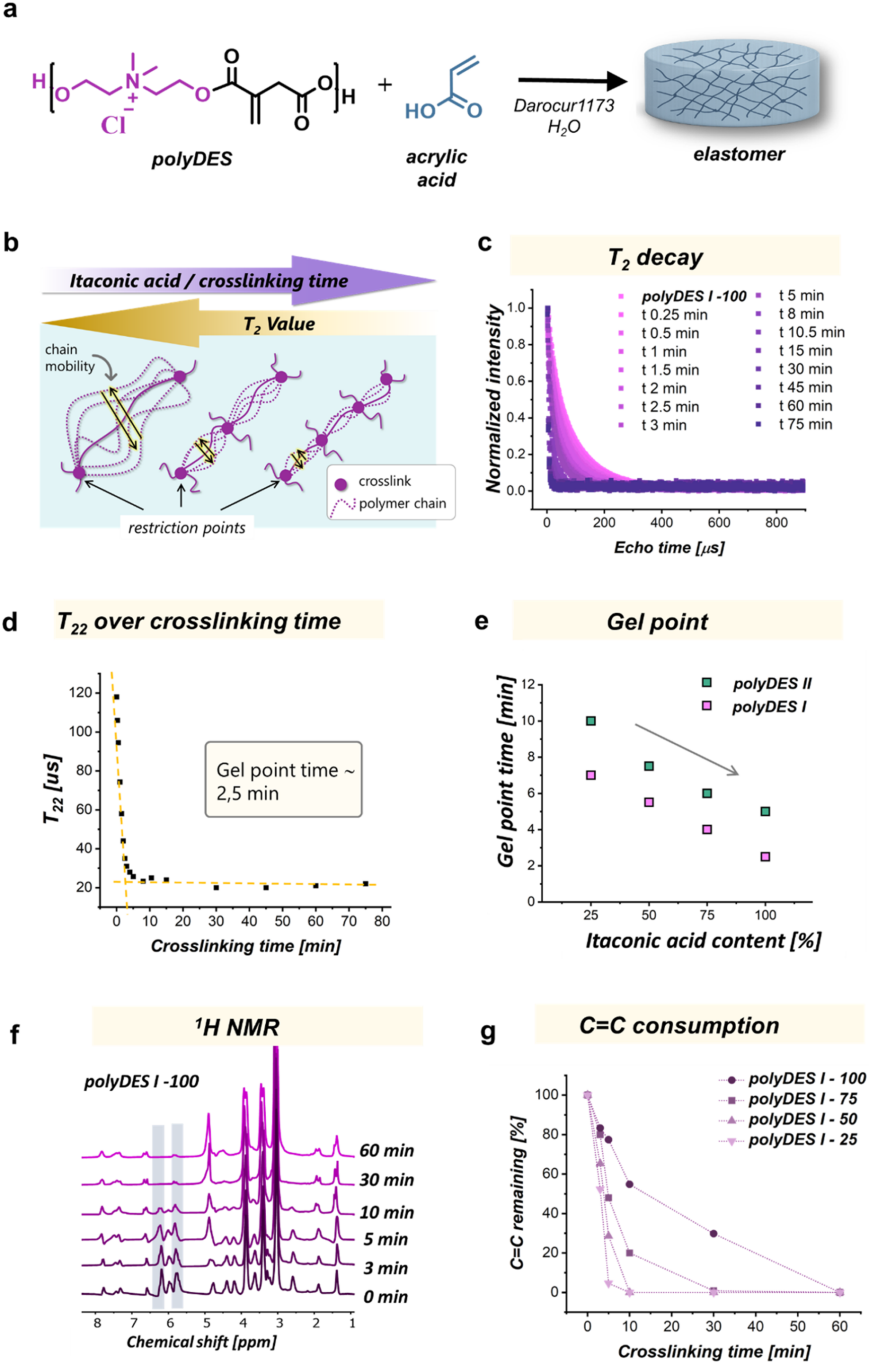
Study of the cationic polyester network during the cross-linking process. (a) Schematic representation of the cross-linking process. (b) Schematic representation of chain mobility and restriction points due to cross-links and their effect on *T*_2_ value. (c) Evolution of *T*_2_ decay over irradiation exposure time of polyDES I-100. (d) *T*_22_ value over the cross-linking time of polyDES I-100. (e) Gel point time over itaconic acid content for polyDES families I and II. (f) ^1^H NMR spectra for different irradiation exposure times of polyDES I-100. The highlighted regions correspond to the protons of vinyl groups from itaconic acid. (g) Evolution of C=C consumption over irradiation time for polyDES I.

Following photo-cross-linking, the materials form transparent, flexible elastomeric films, demonstrating that the polyDES architecture affords homogeneous networks without observable phase separation. This structural uniformity is also reflected in the SEM analysis (Figure S3), which reveals smooth, continuous surfaces and the absence of pores or microstructural heterogeneities, supporting the formation of dense and well-integrated polymer networks.

To gain deeper insight into the network formation and to validate the differences in cross-linking density, the gelation process was monitored through relaxometry, evaluating the evolution of the transverse relaxation time (*T*_2_) as a function of irradiation time up to 75 min (Figure S4a,b). The transverse proton relaxation in polymer networks, measured above their glass transition temperature (*T*_g_), provides insights into both the molecular mobility of the chains and the restrictions imposed by cross-linking points. In this context, differences in the relaxation decays reflect the coexistence of two distinct populations: mobile polymer chains that remain unreacted (sol fraction), which undergo rapid fluctuations and give rise to a slower relaxation component, and chain segments involved in cross-links, whose motion is constrained, producing a faster relaxation component. Figure 3b depicts the evolution of polymer chain mobility during the cross-linking process of itaconic acid–based networks and its correlation with the *T*_2_ relaxation values. At the early stages (left side), polymer chains exhibit high flexibility and large segmental motions, which are represented by wide yellow arrows. These dynamic motions result in slower proton relaxation, corresponding to longer *T*_2_ values. As the cross-linking density increases with irradiation time (moving toward the right), new restriction points (purple nodes) are progressively introduced. These cross-linking points limit the amplitude of chain motions, reducing polymer mobility as indicated by shorter yellow arrows. Consequently, the *T*_2_ decreases, reflecting the transition from a flexible, loosely connected network to a more rigid and constrained polymer structure. For each sample, *T*_2_ was screened for different irradiation times, and each relaxation curve was fitted with a biexponential model, separating the contributions of the rigid (cross-linked) (*T*_21_) and the flexible (sol) (*T*_22_) domains of the polymer (Figures 3c and S4a,b, SI). As the reaction proceeded, the formation of cross-links led to increased rigidity and decreased molecular mobility, which was evidenced by shorter *T*_2_ (*T*_21_ and *T*_22_) values. The evolution of *T*_22_ (associated with the flexible domain) over the cross-linking time is illustrated in Figures 3d and S4, where an initial sharp decrease divides the curve into two regimes. A bilinear regression was used to fit these regions, and the intersection of the two slopes was taken as the gel point time.^22,28^ Figure 3e shows the gel point time for each sample of polyDES families I and II. It was observed that increasing the itaconic acid content significantly accelerates the gelation process in both polyDES families. Additionally, we observed that for the same amount of itaconic acid, the gelation for polyDES I is faster than that for polyDES II. Both trends can be explained by the higher density of vinyl groups per unit mass of polymer, due to either a greater incorporation of itaconic acid or the use of the shorter ionic monomer.

To gain further insights into the cross-linking process, the evolution of C=C consumption under UV exposure was monitored by normalizing the integrated NMR signals of the vinyl protons, where the initial value (100%) corresponds to the total number of double bonds present in the formulation prior to irradiation. Figures 3f and S5 present the spectra of the materials exposed for 3, 5, 10, 30, and 60 min. The highlighted regions correspond to the resonances of the vinyl protons, whose intensity progressively decreases as the irradiation time increases, confirming the consumption of double bonds during cross-linking. This behavior is quantified in Figures 3g and S6, where the relative C=C consumption was determined from the integration of the corresponding NMR signals. In this context, formulations with a lower itaconic acid content (e.g., polyDES I-25 or polyDES II-25) exhibit a more rapid decrease in double-bond proportion. This behavior can be attributed to the lower initial number of reactive sites, which allows the system to reach full conversion more quickly. In contrast, formulations with higher itaconic acid content (polyDES I-100 and polyDES II-100) display slower overall consumption of double bonds. Although these systems contain a larger fraction of reactive groups, the higher cross-linking density generated during irradiation leads to the formation of a dense network, which can restrict radical mobility and hinder effective propagation. The combination of an elevated vinyl group content and diffusional limitations, therefore, results in slower apparent kinetics of C=C consumption.

The relationship between the gelation time and double-bond consumption reveals an interesting compromise for the polyDES networks. As the itaconic acid content increases, the gel point is reached significantly faster, indicating that a critical level of connectivity is rapidly achieved due to the high availability of reactive vinyl groups.^22^ However, this rapid gelation does not translate into an equally fast overall consumption of double bonds. On the contrary, formulations with the highest itaconic acid content exhibit a slight decrease in C=C signals over time, which can be attributed to the formation of a dense cross-linked network that restricts radical diffusion and propagation. Thus, while higher itaconic acid fractions accelerate the onset of gelation, they simultaneously hinder complete vinyl conversion, leaving a larger fraction of unreacted double bonds within the network. This dual effect highlights the complex interplay between chemical functionality and network architecture in dictating the cross-linking kinetics of polyDES elastomers. So, further characterization of the materials was performed by irradiation for 60 min to ensure full consumption of C=C for all materials.

### Evaluation of Network Structure–Property Relationships

The NMR relaxation profiles provide valuable insights into the molecular mobility of the polymer networks. Figure 4a and b shows *T*_2_ decays for polyDES families I and II, respectively, 24 h after cross-linking to let the network relax into a final conformation. The measurements were performed on the elastomers both in their native states, prepared with 20 wt % deionized water, and in their swollen state, obtained by adding 1 mL of D_2_O and allowing the samples to equilibrate for 24 h before analysis. As shown in Figures 4a,b and S7a,b, both sets of measurements exhibit the same overall trend. Samples with a higher itaconic acid content, and therefore a higher cross-linking density, display a more pronounced and rapid decay of the relaxation signal. This is consistent with a reduced chain mobility and a denser network. In contrast, formulations with lower itaconic acid proportions (and therefore lower cross-linking density) exhibit slower relaxation, indicating greater conformational freedom. When the materials are swollen in D_2_O (Figure S7), the relaxation profiles change significantly. The presence of solvent increases the chain mobility and introduces additional dynamic contributions from D_2_O molecules within the network. As a result, the relaxation becomes slower overall compared to the native state. Interestingly, while the influence of cross-linking is still observable (higher itaconic acid content leads to faster relaxation), the differences between formulations are less pronounced in D_2_O than in the dry state.

**Figure 4:**
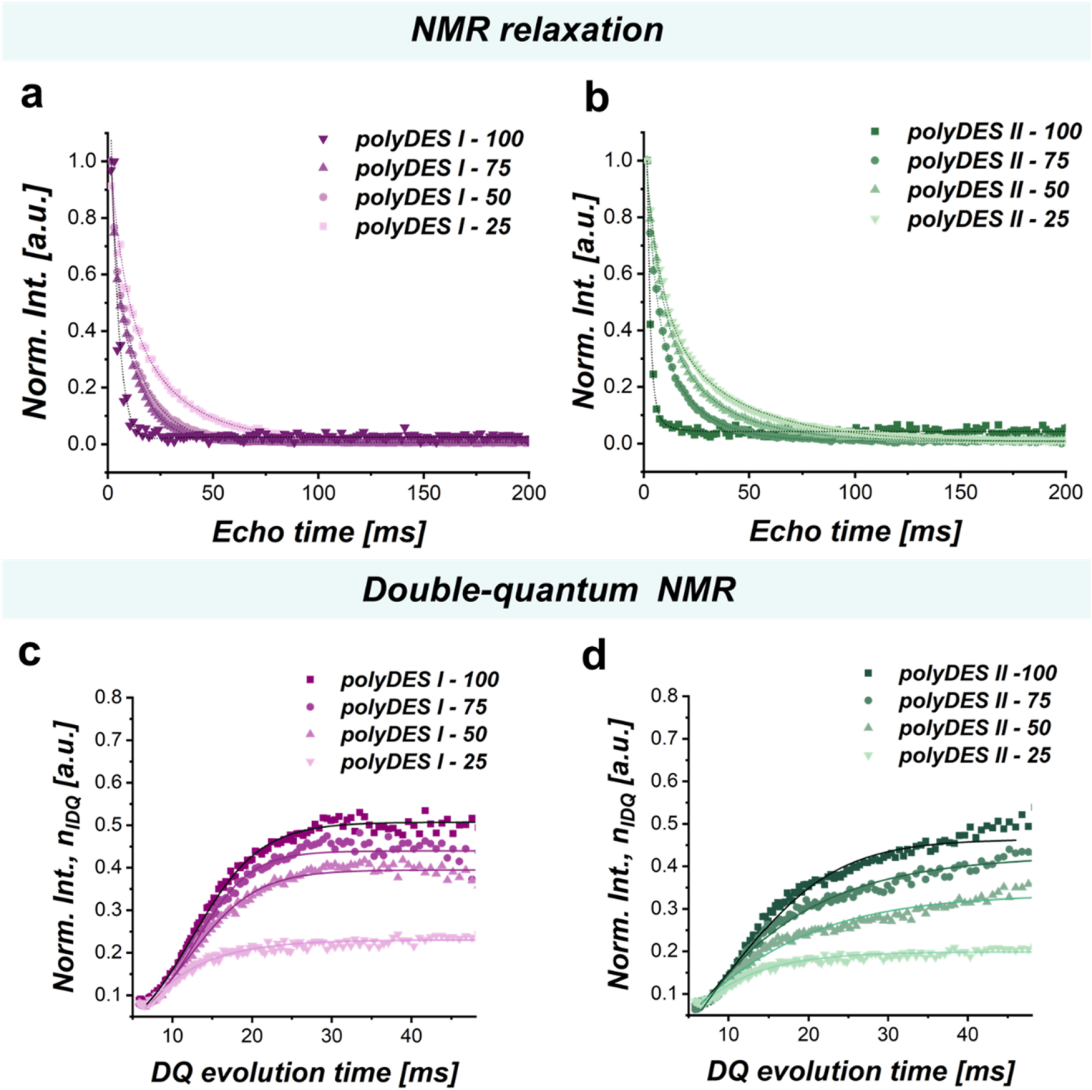
(a, b) Relaxometry characterization by CPMG sequence pulse of elastomers polyDES I and II, respectively. (c, d) Double-quantum evolution curves of polyDES I and II, respectively.

Double quantum (DQ) NMR analysis provides additional information on the architecture of cross-linked polymer networks. In soft materials, residual dipolar couplings accelerate relaxation processes compared to those observed in simple liquids.^29^ The strength of these couplings is inversely related to the average chain length between cross-links, making them a direct indicator of cross-link density.^27,30^ Multiple quantum (MQ) NMR experiments yield a DQ intensity (*I*_DQ_) that scales with both the residual dipolar interaction and the evolution time.^31^ As shown in Figure 4c,d, the normalized DQ build-up curves (*I*_nDQ_) for both polyDES families reveal that samples with higher itaconic/succinic acid ratios exhibit a more pronounced initial build-up. This behavior reflects stronger dipolar couplings, consistent with an increased cross-linking degree and a reduction in the motional freedom of the polymer chains.

To have more insight into the network cross-link over the material behavior, the mechanical tensile properties were evaluated. The mechanical behavior of the cross-linked polyDES networks was strongly influenced by both the relative content of itaconic to succinic acid and the length of the cationic monomer. As shown in Figure 5, polyDES I, containing the shorter cationic monomer, exhibited a much stiffer and more brittle response. Formulations with a higher proportion of itaconic acid (100 and 75) displayed stress–strain curves, reaching maximum stresses in the range of 0.4–0.5 MPa at low deformations, indicative of highly cross-linked and rigid networks. On the contrary, when the fraction of itaconic acid was reduced (50 and 25%), the stress response decreased substantially (<0.2 MPa), and the curves extended to higher deformations (Figure 5a,c), reflecting networks with lower cross-linking density (Figure 5d), enhanced flexibility, and greater deformability before failure.

**Figure 5:**
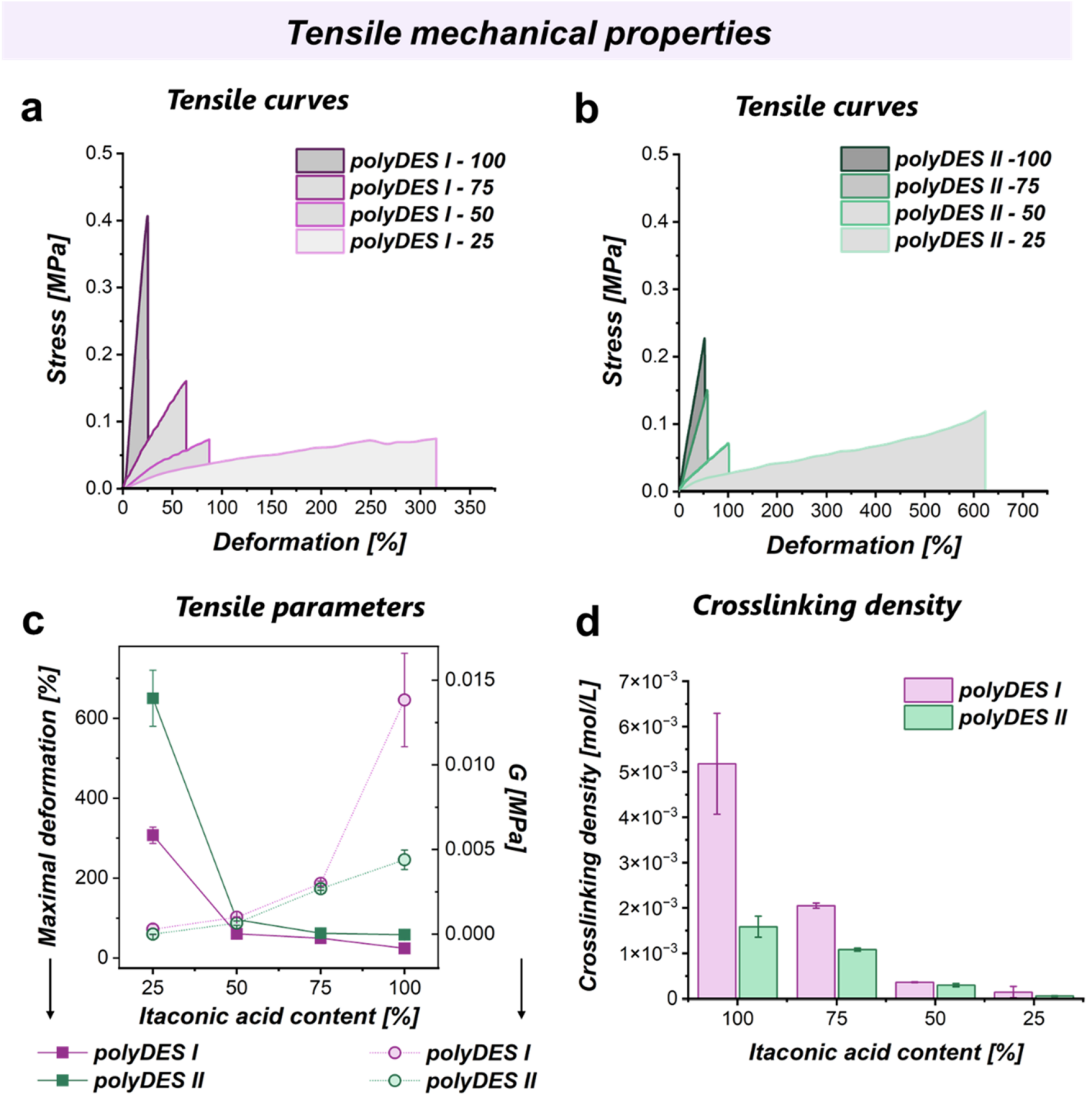
Tensile mechanical properties. (a, b) Stress over deformation of polyDES I and II, respectively. (c) Maximal deformation (dark-filled square points) and *G* modulus (light-filled round points) over itaconic acid content. (d) Cross-linking density over itaconic acid content.

In contrast, polyDES II, based on the longer cationic monomer, showed a markedly different trend (Figure 5b). Although samples with higher itaconic acid contents (100 and 75%) retained a relatively stiffer behavior compared to their succinic-rich counterparts, the maximum stresses were lower than those observed for polyDES I. Importantly, these materials exhibited a significantly enhanced elongation capacity, with some formulations sustaining deformations beyond 600% prior to rupture (Figure 5c). This behavior suggests that the longer cationic monomer introduces additional chain mobility and flexibility, leading to more ductile and extensible networks, even in systems with higher cross-linking potential.

This distinction between brittle and ductile behavior is further illustrated in Figure 5c, where the maximum deformation and shear modulus (*G*) are summarized. PolyDES I exhibited higher *G* values and limited strain at break, especially at elevated itaconic acid contents, consistent with rigid and brittle network structures. On the other hand, polyDES II displayed a clear compensation between modulus and elongation, where reduced stiffness was accompanied by a remarkable increase in extensibility, particularly at intermediate and low itaconic acid levels. Finally, the estimation of the cross-linking density from mechanical data (Figure 5d) provides a direct link to these macroscopic trends. As expected, formulations with higher itaconic acid content presented the highest calculated cross-linking densities, with polyDES I consistently yielding higher values than polyDES II at equivalent compositions. This confirms that the shorter ionic monomer in polyDES I favors the formation of denser networks, whereas the longer ionic monomer in polyDES II allows looser, more flexible architectures, even in the presence of abundant vinyl functionalities. Overall, these results highlight the interplay between the itaconic acid composition and monomer length in dictating the structural and mechanical properties of polyDES.

Assessing the swelling behavior is essential for understanding the interaction of these cationic polyester elastomers with aqueous environments. In networks bearing quaternary ammonium groups, water uptake is particularly relevant because the strong electrostatic interactions between positively charged sites and surrounding water molecules often promote significant hydration and contribute to osmotic swelling. This phenomenon directly impacts mechanical softness, ionic mobility, degradation kinetics, and any prospective biomedical performance, especially in applications in which the material must operate in hydrated or physiological conditions. Therefore, quantifying the swelling response provides key insight into how the ionic content, cross-linking density, and monomer structure collectively dictate the extent of water incorporation in these eutectic-derived elastomeric networks. Figure 6a shows the swelling index over the immersion time. Overall, it can be seen that although these materials are capable of absorbing substantial amounts of water, particularly the more loosely cross-linked formulations, their hydration equilibrium and mechanical resilience clearly distinguish them from conventional hydrogels. Instead, their behavior aligns with water-swellable elastomers, a class of cross-linked polymer networks with rubber-like elasticity that are able to incorporate moderate to significant water into their structure, in this case, driven by the presence of cationic ammonium groups.^32^

**Figure 6:**
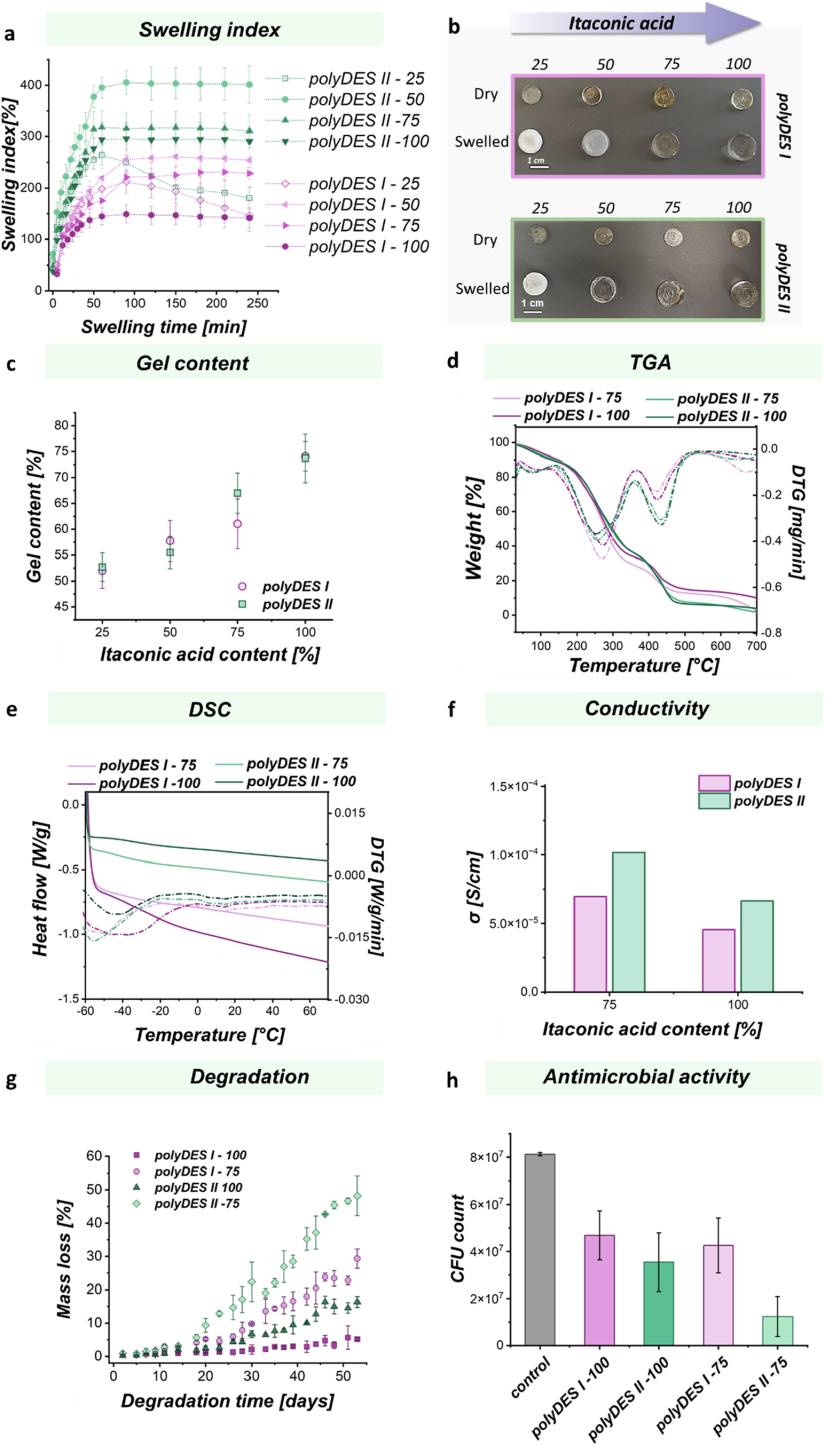
(a) Swelling index in water. (b) Photograph of samples before and after swelling in water. (c) Gel content. (d) TGA analysis. (e) DSC analysis. (f) Conductivity over itaconic acid content. (g) Hydrolytic degradation of polyDES elastomers through mass loss over incubation time. (h) Antimicrobial activity of polyDES elastomers.

Moreover, it was clear that the swelling behavior of the polyDES networks was strongly influenced by the itaconic acid content and the type of ionic monomer used in the formulations. Elastomers prepared with higher amounts of itaconic acid exhibited a marked reduction in swelling capacity, which is consistent with the increased cross-linking density. This effect was more pronounced for polyDES II, where the swelling index remained significantly higher across all compositions, reflecting differences in network structure and chain flexibility compared to polyDES I. Interestingly, formulations containing 25% itaconic acid began to lose material after approximately one h of swelling, suggesting that the polymer network was not fully formed or mechanically stable at this composition. The likely low molar mass of the formed polyDES could compromise the integrity of the swollen network at the lowest itaconic acid content. This observation is in line with the photographic comparison showing a white and opaque film in comparison to a more compact and transparent material after hydration for higher itaconic acid content (Figure 6b). The gel content further supports these findings (Figure 6c), confirming that a reduced fraction of insoluble material for lower itaconic acid content correlates with a weaker network integrity. Collectively, these results highlight that the balance between itaconic and succinic acid not only dictates the extent of chemical cross-linking but also governs the water uptake capacity and physical stability of the resulting networks. Based on these findings and the lack of network integrity in an aqueous environment of the films with lower itaconic acid content, we selected only polyDES I-75/100 and polyDES II-75/100 for further studies.

Thermal properties were evaluated by thermogravimetric analysis (TGA) and differential scanning calorimetry (DSC). The TGA profiles (Figure 6d) showed a multistep degradation behavior characteristic of cross-linked polyesters, with an initial weight loss below 150 °C associated with residual moisture and volatile compounds, followed by major decomposition stages between 250 and 450 °C. DSC thermograms (Figure 6e) revealed *T*_g_ values in the subzero region for all formulations falling within the range expected for elastomeric materials, which are defined by the presence of a glass transition significantly below room temperature to ensure rubber-like behavior under ambient conditions. Within this elastomeric regime, polyDES II exhibited lower *T*_g_ values compared to polyDES I at equivalent itaconic acid compositions (Figure S8, SI). For the 75% itaconic acid formulations, *T*_g_ decreased from −43 °C (polyDES I) to −50 °C (polyDES II), while for the 100% itaconic acid samples, *T*_g_ dropped from −30 °C (polyDES I) to −35 °C (polyDES II). This indicates that polyDES II possesses greater chain mobility and reduced rigidity compared to polyDES I. Moreover, the increase in *T*_g_ with a higher itaconic acid content within each series suggests a more constrained network at higher unsaturation.

The ionic conductivity measurements (Figure 6f) further highlight the structural differences between the two polyDES series. For comparison reasons, all samples were prepared by weight, assuming that each elastomer network retained a similar amount of water under the measurement conditions. This normalization allows the observed differences in conductivity to be attributed primarily to intrinsic structural features rather than variations in the water content. The conductivity results reveal a nonintuitive trend when comparing the two series of elastomers. In principle, polyDES I, prepared from a shorter ionic monomer, is expected to contain a higher concentration of ionic sites per unit mass of the polymer film. Such an increase in the charge density should favor ionic transport. However, the experimental data show the opposite behavior with polyDES II consistently exhibiting higher conductivity values, regardless of the itaconic acid content. This discrepancy suggests that the ionic charge density is not the dominant factor governing conductivity in these systems. Instead, the results point to the critical role of the network structure and segmental dynamics. PolyDES I presents higher stiffness, indicative of a denser and more tightly cross-linked network. Such features restrict segmental motion, reduce the available free volume, and increase the tortuosity for ion transport, thereby limiting ion mobility. In contrast, polyDES II, with its longer ionic spacer, displays lower network rigidity, conditions that enhance polymer chain flexibility and water uptake. These characteristics favor the solvation and mobility of ions, leading to higher conductivities observed despite the lower intrinsic ionic site density. Furthermore, the increase in itaconic acid content, associated with a higher cross-linking degree, results in a reduction of conductivity in both series. This trend reinforces the conclusion that ion mobility, modulated by network softness, exerts a stronger influence than the nominal concentration of charged groups.

The degradation behavior of polymeric networks plays a crucial role in determining their suitability for biomedical applications. Controlled degradation ensures that the material can gradually disassemble or resorb after fulfilling its function, avoiding chronic inflammation and the need for surgical removal. Moreover, the degradation rate can be tuned to match tissue regeneration kinetics or drug release profiles, enabling time-dependent therapeutic performance. Therefore, assessing the hydrolytic stability of these cationic networks provides valuable information for predicting their long-term behavior in physiological conditions and for designing materials with applications that require specific degradation rates. Figure 6g shows the hydrolytic degradation through mass loss over the incubation time. At early stages (up to ∼15 days), all samples exhibited minimal mass loss (<5%), indicating that the networks are initially stable and resistant to hydrolytic erosion. Beyond this lag period, the degradation rate increased steadily, consistent with a bulk hydrolysis mechanism in which water penetration precedes chain scission. In this stage, distinct differences become evident between polyDES I and polyDES II formulations.

Elastomers derived from polyDES II show the highest degradation rates, particularly the formulation with 75% itaconic acid, which undergoes a rapid and almost linear mass loss after ∼20 days, reaching values close to 50% at 54 days. This pronounced susceptibility can be explained by the lower cross-linking density at 75% itaconic acid, which results in a more open network structure, facilitating water diffusion and hydrolytic cleavage. In contrast, the 100% itaconic acid polyDES II elastomer, while still more degradable than its polyDES I counterpart, exhibits a slower mass loss profile due to the higher degree of cross-linking that stabilizes the structure. Overall, the results reveal a clear structure–property relationship: shorter ionic monomers (polyDES I) promote more compact networks with enhanced resistance to degradation, while longer monomers (polyDES II) result in looser, more hydrated structures that are prone to faster mass loss. Furthermore, decreasing the itaconic acid content reduces cross-linking density and accelerates degradation in both systems. This dual effect highlights how tuning both the ionic monomer design and the cross-linking composition provides a powerful strategy to control film lifetime and degradation kinetics, which is crucial for tailoring them to specific applications such as drug delivery or wound healing.

Polymers containing cationic functionalities are of particular interest for biomedical applications, as the presence of charged moieties can endow the material with inherent antimicrobial properties. Such activity is highly desirable in systems intended for wound dressings, tissue scaffolds, or drug-controlled release, where microbial contamination may compromise the functionality and biocompatibility. In this context, the antimicrobial potential of the polyDES elastomers was assessed to explore their suitability for biorelated uses. The assays were performed against *E. coli*, a Gram-negative model bacterium commonly employed to evaluate antibacterial performance due to its well-characterized physiology and relevance as an opportunistic pathogen in biomedical environments. The outer membrane of *E. coli*, rich in negatively charged lipopolysaccharides, also provides an appropriate model to probe the effectiveness of electrostatic and contact-based bactericidal mechanisms driven by cationic polymeric surfaces.^33^

The antimicrobial contact assay revealed a clear difference among the four elastomer formulations, following the order of activity: polyDES II-75 > polyDES II-100 ≈ polyDES I-75 ≈ polyDES I-100. The strongest inhibitory effect was achieved for polyDES II-75, while the remaining samples exhibited moderate and comparable antibacterial performance (Figure 6h). This trend emphasizes that the antimicrobial activity is not solely determined by the nominal ionic charge density of the network but rather by the interplay between network architecture, chain mobility, and accessibility of cationic sites. Although polyDES I, containing the shorter ionic monomer, possesses a higher theoretical charge density per mass of polymer, its denser and more rigid network restricts the exposure of quaternary ammonium groups. In contrast, polyDES II, derived from the longer cationic spacer, forms more flexible and hydrated networks that facilitate the diffusion of water and the outward orientation of charged moieties. These structural characteristics enhance electrostatic interactions with negatively charged bacterial membranes, promoting possible membrane disruption. The pronounced effect of polyDES II-75 can be attributed to its lower cross-linking density and higher swelling degree, which increase chain mobility and surface accessibility of functional groups. In comparison, the more densely cross-linked polyDES II-100 presents reduced flexibility, limiting the dynamic contact with bacterial cells and slightly decreasing antimicrobial efficiency. This behavior aligns with previous reports showing that films with lower cross-linking density often display enhanced antimicrobial activity due to increased chain mobility and exposure of active groups.^34,35^

## Conclusions

This work demonstrates the potential of monomeric eutectic mixtures as an effective platform for the design of sustainable and functional elastomers. By combining ChCl–based ionic diols with bioderived diacids such as itaconic and succinic acid, it was possible to synthesize cationic polyester networks. The straightforward synthesis and the liquid nature of the eutectic monomers enabled the preparation of networks with tunable composition through a mild polycondensation–photo-cross-linking strategy. This approach highlights the versatility of DEM chemistry, which simplifies synthesis under mild conditions while enabling the development of biodegradable and environmentally friendly materials.

The resulting polyDES elastomers exhibit a strong relationship between molecular design and macroscopic performance, where monomer architecture and acid composition govern mechanical, thermal, swelling, ionic transport, and degradation behaviors. Notably, the combination of cationic functionality and hydrolytically degradable polyester backbones is rare among synthetic elastomers, since ionic polymers often rely on stable, nondegradable frameworks. The ability to integrate biodegradability into cationic networks represents a significant step toward transient and resorbable materials suitable for biomedical applications.

In addition, a remarkable outcome of this study is the inherent antimicrobial activity exhibited by the cationic polyester elastomer, particularly by polyDES II-75, which showed the strongest inhibition against *E. coli*. This effect is attributed to the synergistic influence of network softness, swelling capacity, and exposure of cationic sites, which together enhance electrostatic interactions with bacterial membranes. These results demonstrate that antimicrobial efficiency can be optimized not only through charge density but also by tailoring the network architecture and chain mobility.

Overall, this study underscores the versatility of eutectic-derived chemistry as a sustainable route to develop biodegradable, antimicrobial, and ionically conductive cationic elastomers, expanding their potential for biomedical applications, such as wound healing, drug delivery, and tissue regeneration.

## Supplementary Material



## Notes

Monomer synthesis and ^1^H NMR spectra (Figure S1); theoretical monomer content (Table S1); water vapor uptake measurements (Figure S2); SEM micrographs (Figure S3); NMR relaxation analysis during cross-linking (Figures S4 and S7); ^1^H NMR after UV irradiation and C=C consumption (Figures S5 and S6); and glass transition temperatures (Figure S8) (PDF)
