# Supplementary material for: Degradable Water-Swellable Elastomers from Biobased Deep Eutectic Monomers
Source: ACS Sustain Chem Eng. 2026 Mar 31;14(14):6704–18. doi: 10.1021/acssuschemeng.5c13563 (PMC13081223; doi:10.1021/acssuschemeng.5c13563)
Supplement: Supplementary file 1 [file sc5c13563_si_001.pdf]

Supporting Information (SI)

Degradable Water-Swellable Elastomers from Bio-based Deep Eutectic Monomers

Lucila Navarro[1]*, Matías D. Hartman[2], Sebastian Locatelli[3], Santiago E. Vaillard[1], Matías L. Picchio[4,5]*, Roque J. Minari[3], Haritz Sardon[6] and David Mecerreyes[5,7]

[1] Group of Applied Organic Chemistry, Instituto de Desarrollo Tecnológico para la Industria Química (INTEC), CCT-Santa Fe, CONICET-UNL, Colectora Ruta Nacional. 168, Km 1, Paraje EL Pozo, Santa Fe 3000, Argentina

[2] Universidad Nacional del Litoral (UNL), Facultad de Bioquímica y Ciencias Biológicas, Edificio FBCB, Ciudad Universitaria UNL, C.C. 24, (S3000ZAA), Santa Fe, Argentina

[3] Group of Polymers and Polymerization Reactors, Instituto de Desarrollo Tecnológico para la Industria Química (INTEC), CCT-Santa Fe, CONICET-UNL, Colectora Ruta Nacional. 168, Km 1, Paraje El Pozo, Santa Fe 3000, Argentina

[4] POLYMAT, Department of Mining-Metallurgy Engineering and Materials Science, School of Engineering, University of the Basque Country (UPV/EHU), Plaza Torres Quevedo 1, 48013 Bilbao, Spain

[5] IKERBASQUE, Basque Foundation for Science, Plaza Euskadi 5, Bilbao, 4009 Spain

[6] POLYMAT, Department of Polymers and Advanced Materials: Physics, Chemistry and Technology, Faculty of Chemistry, University of the Basque Country UPV/EHU, Paseo Manuel de Lardizábal, 3, 20018 Donostia-San Sebastián, Spain

[7] POLYMAT, University of the Basque Country UPV/EHU, Avenida Tolosa 72, Donostia-San Sebastián, Gipuzkoa 20018, Spain

Corresponding authors:

lnavarro@intec.unl.edu.ar

matiasluis.picchiop@ehu.eus

Number of pages: 11

Number of figures: 8

Number of tables: 1

Content

Figure S1. a) Monomer synthesis by quaternization reaction. b) ^{1}H NMR of obtained monomers....3

Table S1. Theoretical monomer content for each polyDES 4

Figure S2. Water Vapor uptake of polyDES I and polyDES II as a function of itaconic acid content (25–100%). Measurements were performed after incubating dry elastomer films in a sealed chamber at 75% relative humidity until reaching mass equilibrium (7 days). 5

Figure S3. SEM micrographic pictures: Surface view and transversal cut. 6

Figure S4. a) and b) Normalized intensity decay (upper panel) and the T_{22} value over crosslinking time (down panel)for polyDES family I and II, respectively. 7

Figure S5. ^{1}H NMR spectra for polyDES I and II after different UV irradiation times......................8

Figure S6. C=C consumption after different irradiation periods. 100% corresponds to the integration of the C=C-$_{H2}$ signal of the blend (prepolymer and acrylic acid) 9

Figure S7. Normalized T_2 relaxation decay of swelled in D_2O elastomers using CPMG pulse sequence for a) polyDES I and b) polyDES II...10

Figure S8. Tg values for elstomers polyDES I and II, in 75 and 100 % content of itaconic acid.....11

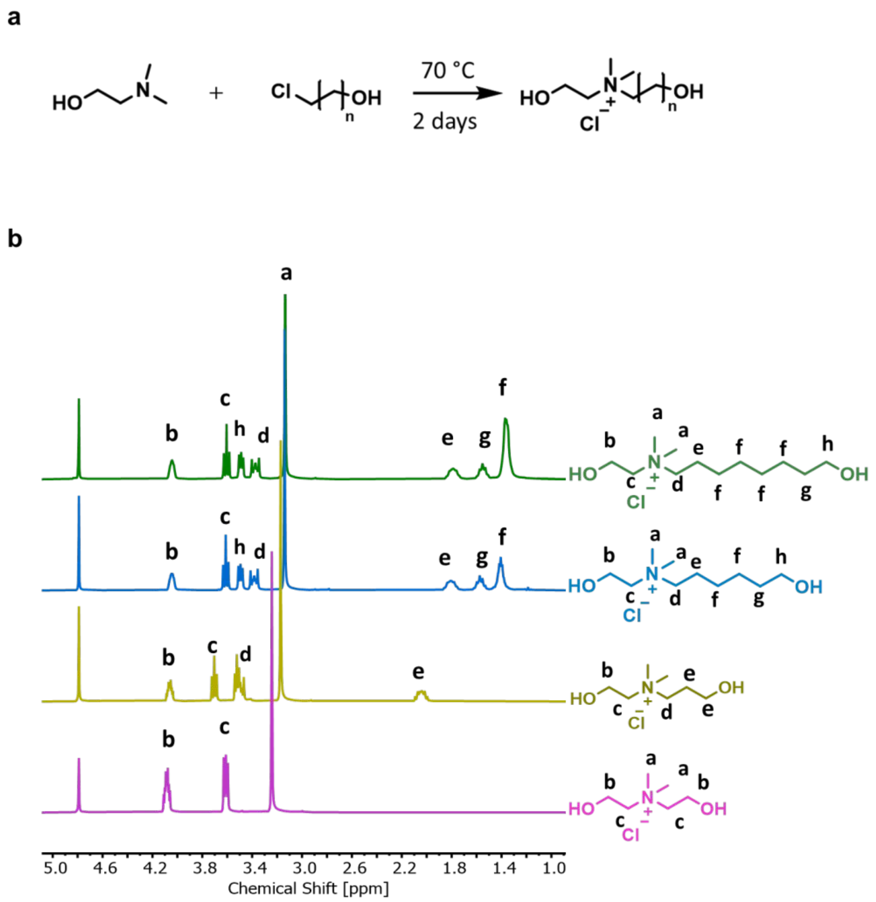

Figure S1. a) Monomer synthesis by quaternization reaction. b) ^{1}H NMR of the obtained monomers.

Table S1. Theoretical monomer content for each polyDES

**PolyDES code**	***MA***	***MD***	**Itaconic acid**	**Succinic acid**
PolyDES I – 100	100	0	100	0
PolyDES I – 75			75	25
PolyDES I – 50			50	50
PolyDES I – 25			25	75
PolyDES II – 100	0	100	100	0
PolyDES II – 75			75	25
PolyDES II – 50			50	50
PolyDES II – 25			25	75

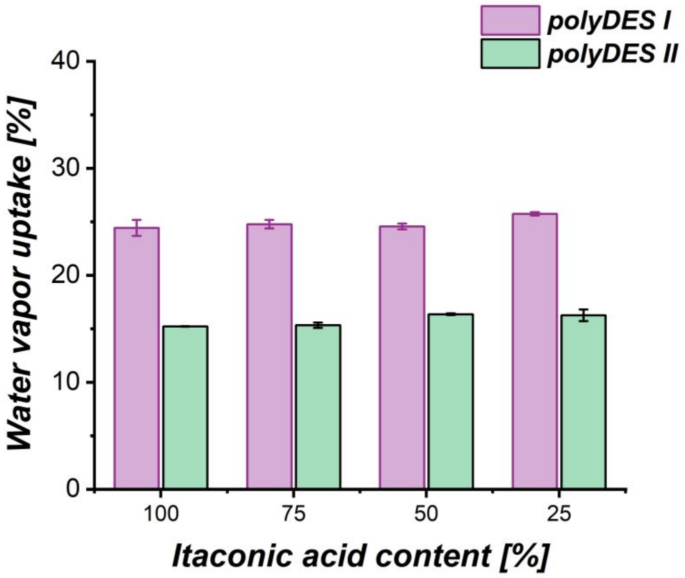

Figure S2. Water Vapor uptake of polyDES I and polyDES II as a function of itaconic acid content (25–100%). Measurements were performed after incubating dry elastomer films in a sealed chamber at 75% relative humidity until reaching mass equilibrium (7 days).

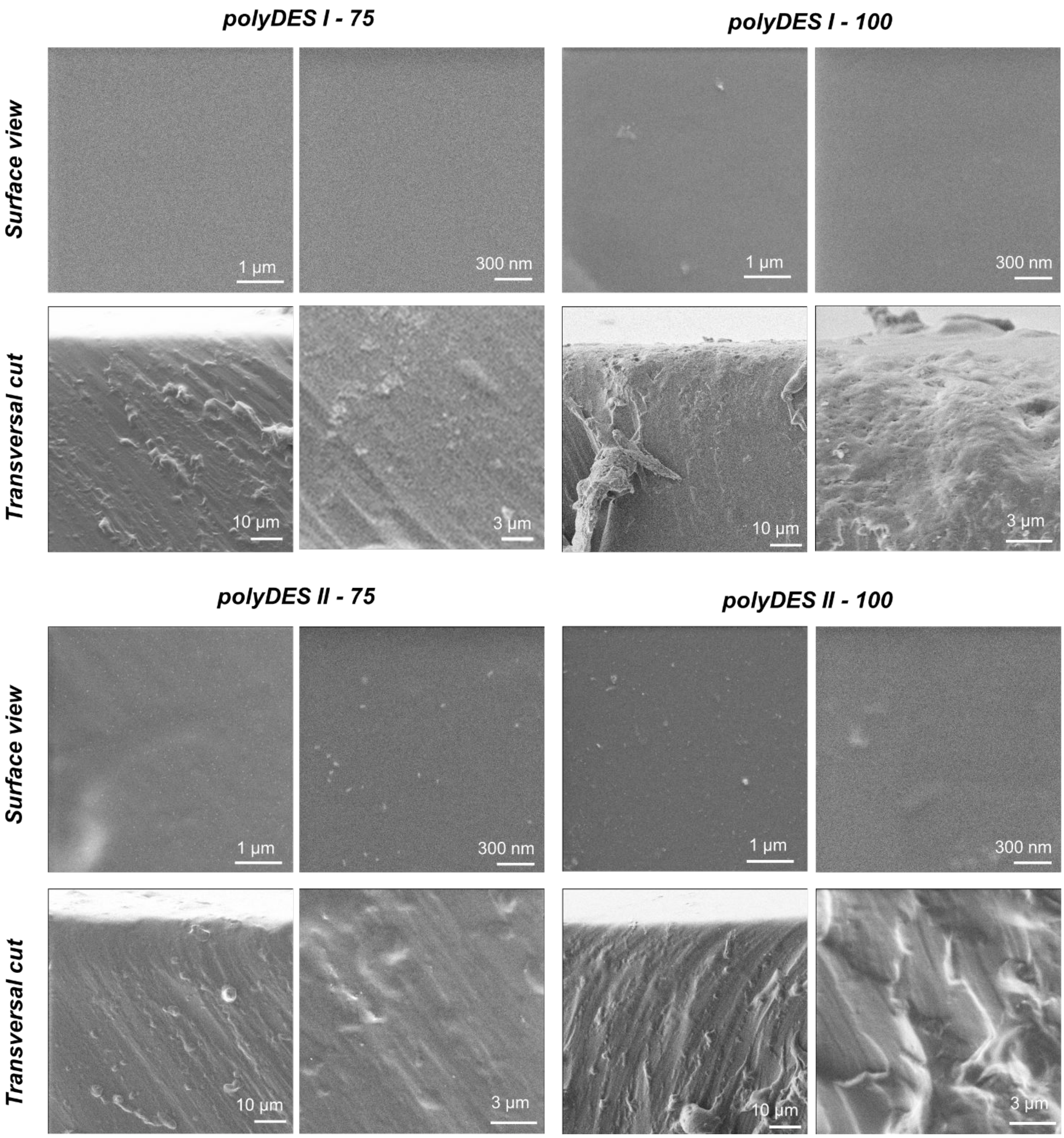

Figure S3. SEM micrographic pictures: Surface view and transversal cut.

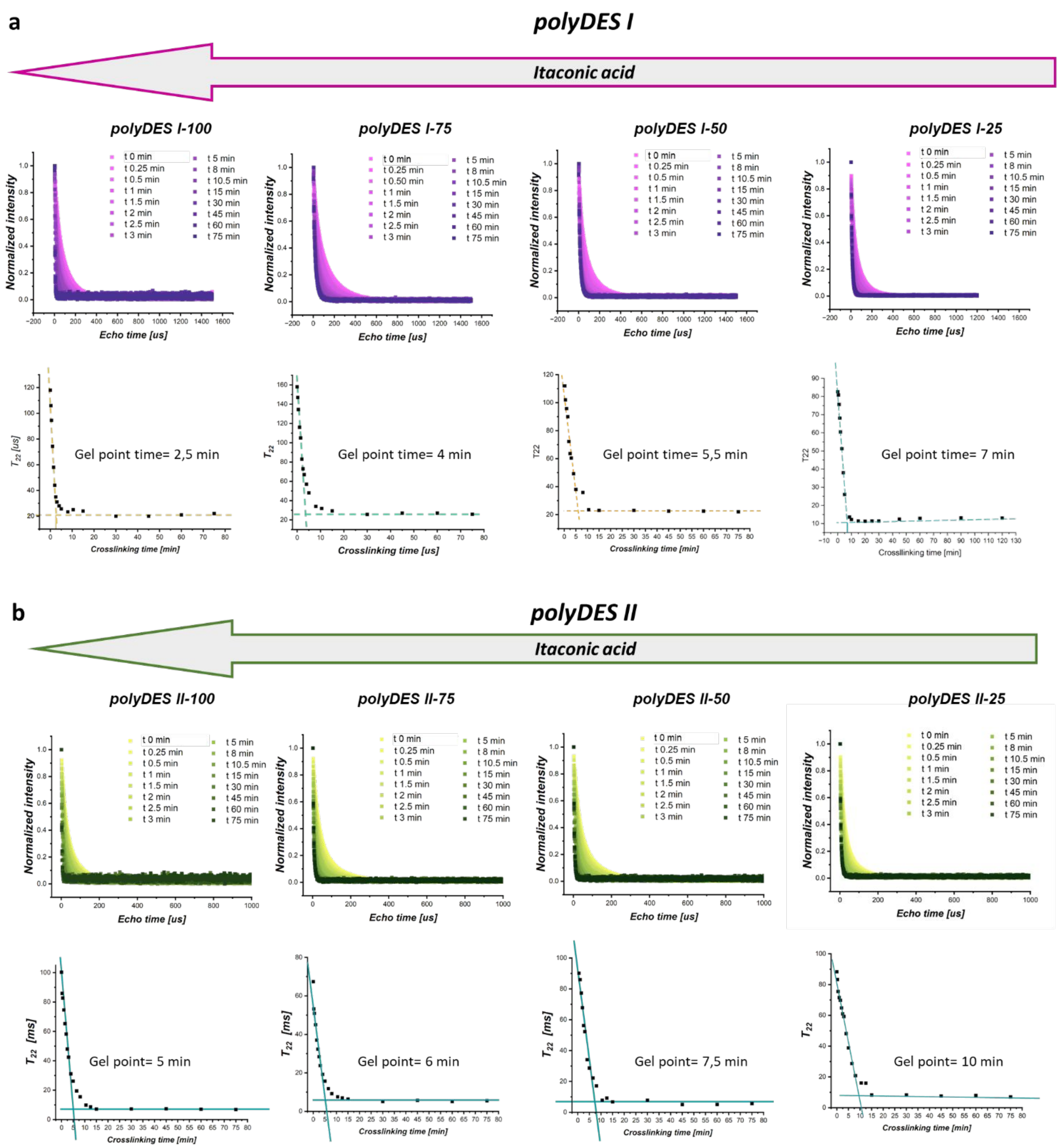

Figure S4. a) and b) Normalized intensity decay (upper panel) and the T_{22} value over crosslinking time (lower panel)for polyDES family I and II, respectively.

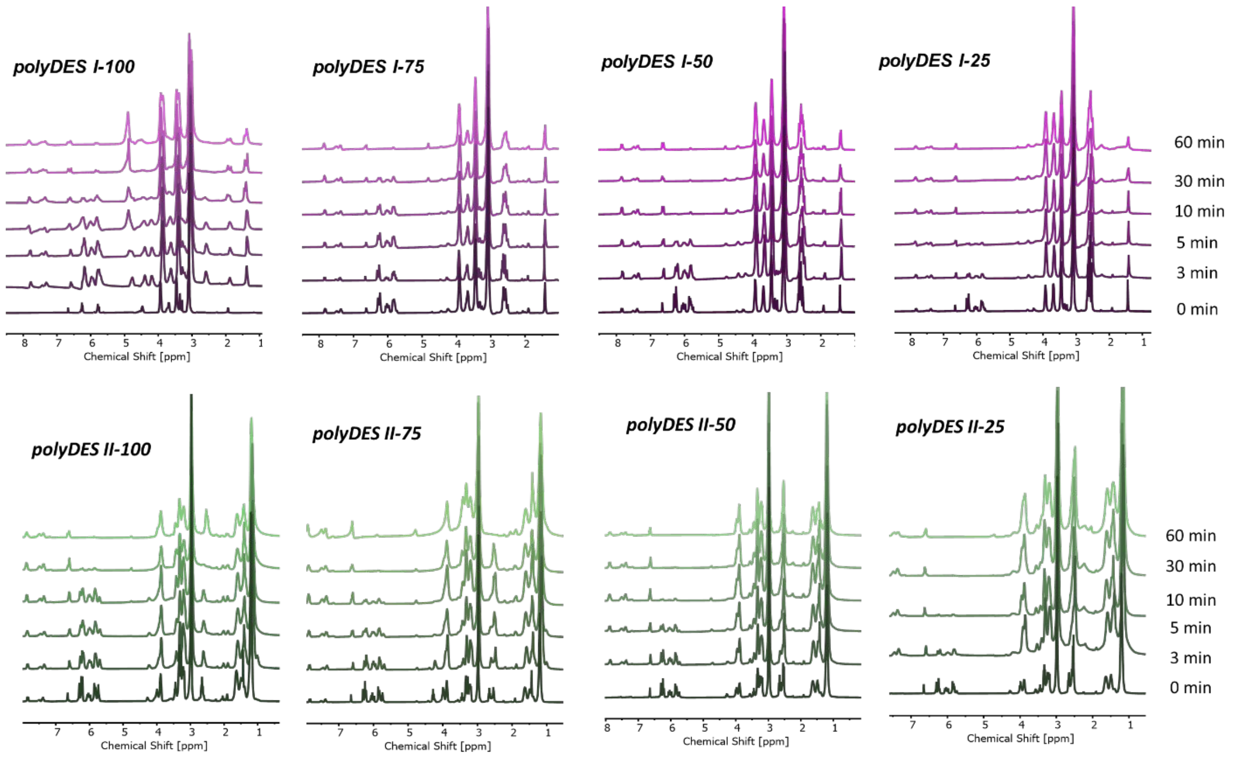

Figure S5. ^{1}H NMR spectra for polyDES I and II after different UV irradiation times.

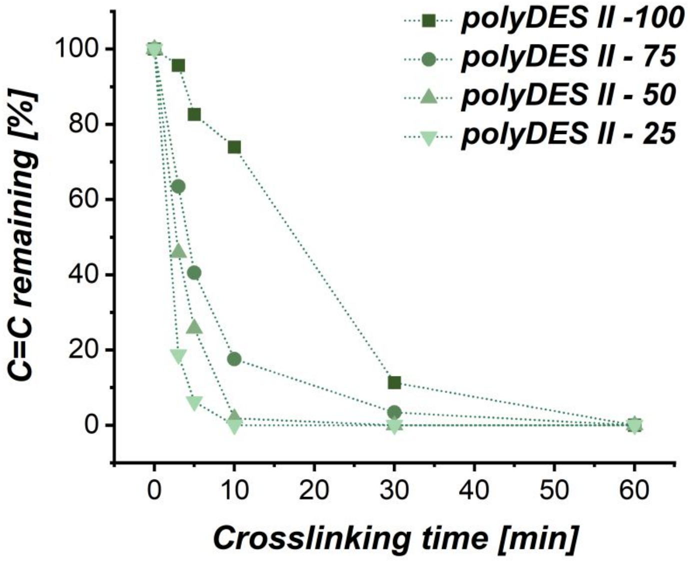

Figure S6. C=C consumption after different irradiation periods. 100% corresponds to the integration of the C=C-$_{H2}$ signal of the blend (prepolymer and acrylic acid)

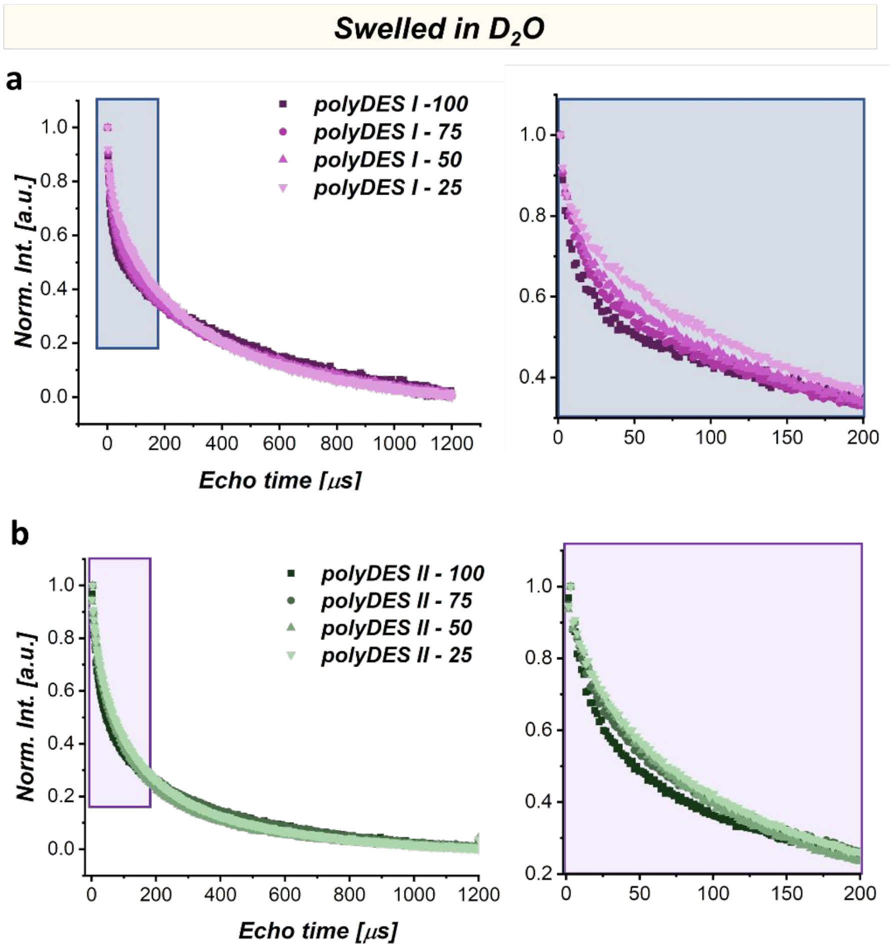

Figure S7. Normalized T_2 relaxation decay of swelled in D_2O elastomers using CPMG pulse sequence for a) polyDES I and b) polyDES II.

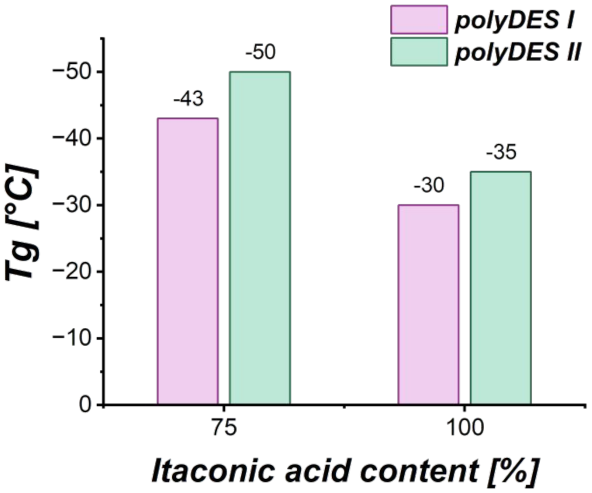

Figure S8. Tg values for elastomers polyDES I and II, in 75 and 100 % content of itaconic acid.
